# Comparative efficiency of silica gel, biochar, and plant growth promoting bacteria on Cr and Pb availability to *Solanum melongena* L. in contaminated soil irrigated with wastewater

**DOI:** 10.3389/fpls.2022.950362

**Published:** 2022-08-04

**Authors:** Umm e Rabiya, Muhammad Ali, Muhammad Ansar Farooq, Zafar Siddiq, Saud A. Alamri, Manzer H. Siddiqui, Waqas-ud-Din Khan

**Affiliations:** ^1^Sustainable Development Study Centre, Government College University, Lahore, Pakistan; ^2^Institute of Environmental Sciences and Engineering, School of Civil and Environmental Engineering, National University of Sciences and Technology, Islamabad, Pakistan; ^3^Department of Botany, Government College University, Lahore, Pakistan; ^4^Department of Botany and Microbiology, College of Science, King Saud University, Riyadh, Saudi Arabia; ^5^Tasmanian Institute of Agriculture, University of Tasmania, Hobart, TAS, Australia

**Keywords:** chitosan polymerized silica, microbial metal removal efficiency, metal translocation factor, defense mechanism of plant, principal component analysis

## Abstract

Crop irrigation with untreated wastewater is a routine practice in developing countries that causes multiple human health consequences. A comparative study was performed to regulate total Cr and Pb stress in soil and *Solanum melongena* L. plant. For this purpose, 0.2% chitosan polymerized silica gel (CP-silica gel), 1.5% zinc-enriched biochar (ZnBc), and three bacterial species such as *Trichococcus* sp. (B1), *Pseudomonas alcaligenes* (B2), and *Bacillus subtilis* (B3) were selected. Initially, a biosorption trial was conducted to test the heavy metal removal efficiency of three bacterial species B1, B2, and B3 for 24 h. Hence, B3 showed maximum Cr and Pb removal efficiency among the studied bacterial isolates. Then, a pot study was conducted with 12 different treatments having three replicates. After harvesting, different growth and biochemical parameters such as chlorophyll concentration, proteins, phenolics, reactive oxygen species, and antioxidant enzymes were analyzed. The results demonstrated that wastewater application significantly (*p* ≤ 0.01) reduced the fresh and dry weights of the root, stem, and leaves due to high total Cr and Pb toxicity. However, CP-silica gel and ZnBc treatments performed best when applied in combination with B3. The concentration of leaf total Cr was significantly decreased (91 and 85%) with the application of ZnBc + B3 and CP-Silica gel + B3, respectively, as compared to control. There was a reduction in stem hydrogen peroxide (87%) and malondialdehyde (81%) recorded with CP-silica gel + B3 treatment due to enhanced activities of antioxidant enzymes viz. ascorbate peroxidase (6-folds) and catalase (7-folds) relative to control. Similarly, leaf total phenolics (3-folds) and protein (6-folds) contents were enhanced with CP silica gel+B3 application relative to control. Overall, CP-silica gel and ZnBc with B3 application proved to be the most appropriate treatments and can be used in developing countries to limit the deleterious effects of total Cr and Pb pollution.

## Introduction

Direct discharge of tanneries wastewater into the environment causes an increase in the level of soil heavy metals (HMs) stress especially chromium (Cr^6+^) and lead (Pb^2+^), and thus poses a serious limitation to achieving sustainable development and food security (Elahi et al., 2018; Farid et al., 2018). In Pakistan, almost 80% of wastewater is being used for irrigation purposes which increases the HMs concentrations inside the crops and vegetables; hence, carcinogenic health issues in humans mostly occur after consuming those crops/vegetables (Sandeep et al., 2019; Khalilzadeh et al., 2020).

Chromium (VI) and Pb^2+^ are highly toxic metals that hinder plant growth and development and cause severe diseases in humans through bioaccumulation and biomagnification (Turan et al., 2018a; Suliman et al., 2020). Heavy metal toxicity causes oxidative stress in plants by producing excess amounts of reactive oxygen species (ROS) by suppressing its antioxidant enzymes and other metabolites (Suliman et al., 2020). Plants show various Cr^6+^ toxicity symptoms such as retardation in growth and development, alterations of germination processes, and reduced growth of roots, leaves, and stems. So, all these factors cumulatively cause compromised crop productivity and yield losses (Hasanuzzaman et al., 2020). Likewise, Pb^2+^ can easily enter the roots of plants through different ionic channels where it can further be translocated by following the water channels (xylem) and causes severe damage to cellular constituents (Dad et al., 2021). In general, different methods such as bioremediation and application of organic or inorganic substances are employed to counter the deleterious effects of metal toxicity in the rhizosphere; however, the combined application of one or more detoxifying methods could pronounce the positive impacts on plant growth under unfavorable conditions.

As an inorganic amendment, a hybrid of chitosan and silica gel through polymerization (CP-silica gel) acts as a strong adsorbent of heavy metals (Fouad and Ghanem, 2017). In an acidic medium (pH 5–6.5), the adsorption capacity of CP-silica gel is higher than the alkaline medium (Radi et al., 2020). A study by Sharma et al. (2019) described that silica gel improves plant defense against external attack by forming an inner protective shell. Similarly, chitosan is also reported as an effective source to scavenge ROS from plants (Khan et al., 2018). CP-silica bio-gel (encapsulation of microbial species with CP-silica gel) material helps to degrade a spectrum of pollutants; hence, balancing the structure and functions of the ecosystem (Sakloos et al., 2017).

Similarly, biochar application against HMs stress proved to be a good strategy; but in recent years, it was established that the adsorption capacity of biochar efficiency increases when it is doped/modified by some mineral nutrients (Wang et al., 2020). There are many positive effects of doped biochar reported in literature like it reduces the HMs uptake in plants; electrical conductivity and pH of soil were also improved by its application (Almaroai et al., 2014). Another study about the effectiveness of doped biochar showed that applications of biochar (enriched with Fe, Si, and Zn) stimulate crop growth and yield by triggering the activities of SOD (superoxide dismutase), POD (peroxidase dismutase), CAT (catalase), and APX (ascorbate peroxidase) in Cr^3+^, Cu^2+^, and Pb^2+^ contaminated soil (Yu et al., 2020). Micro-porous structure of carbon is activated by ZnCl_2_ so zinc-enriched biochar is a useful amendment for eco-friendly growth and development of plants in Cr^3+^-contaminated soils (Duan et al., 2019).

Microbial application in metal-contaminated soil to avoid soil/plant toxicity has been reported as a viable approach as almost 60–80% of heavy metals are removed by bacterial species (Wei et al., 2020). However, Lan et al. (2021) reported that the efficiency of bacteria in detoxifying the Cr^6+^, Pb^2+^, Zn^2+^, and Cu^2+^ stress could be enhanced when applied in combination with pinecone-based biochar. A study by Li et al. (2022) investigated that availability of HMs (Pb, Cd, and Zn) can be effectively reduced (Pb: 51.25%, Cd: 26.25%, and Zn: 34.25%) by the coexistence of bacterial species (*Pseudomonas, Bacillus, Lysobacter*, and *Brevundimonas*), Zn-doped biochar, and carbon aerogel nanoparticles. Another study by Wang et al. (2020) reported that the growth of pakchoi vegetable was significantly enhanced (>18%) by applying silicon fertilizer (0.8–3.2%) and its association with soil bacterial species which reduced the content of HMs (Pb, Cu, Hg, Zn, Cd, Cr, and Co) in soil. Literature is available about applications of Zn-enriched biochar and CP-silica gel as discussed earlier but their interaction with microbes to regulate tanneries wastewater stress for the plant growth remains a big question.

So, this study aimed to fulfill the gaps in scientific research by creating a correlation of microbes with organic and inorganic adsorbents. These might efficiently regulate heavy metal stress in a more innovative and eco-friendly way to meet the goals of sustainable development. More specifically, we tend to examine the deleterious effects of tanneries wastewater on different physiological and biochemical parameters of *Solanum melongena* L. plant, which further evaluate the efficiency of Zn-enriched Bc and CP-silica gel when combined with different types of bacterial sp. to regulate the heavy metal stress in soil and plant. However, it was expected that the application of CP-silica gel, ZnBc, and bacterial sp. might efficiently enhance the physiological and biochemical parameters of the *Solanum melongena* L. plant by regulating the effect of heavy metals stress in soil.

## Materials and Methods

### Wastewater sampling scheme

Wastewater slurry was collected from Siddiq Leather Works (Pvt) Ltd., located at Lahore Sheikhupura Road, Pakistan at 31°37′48.02"E and 74°13′01.25"N. Then, the slurry was passed through the filtration setup (through Whatman filter paper) to remove any types of solids. The concentrations of different heavy metals were estimated by preparing the different formulations of that slurry (10, 20, 30, 50, 75, and 100%) by diluting it with distilled water (Supplementary Figure S1; Table 1). The diluted concentrations of heavy metals were measured by using Atomic Absorption Spectrophotometer (ISE-3000 series, Thermo Fischer, United States) (which used acetylene gas for analysis) and formulation with 30% slurry (in which concentrations of Cr and Pb were 291.83 and 4.795 mg L^−1^, respectively) was chosen for the pot experiment as wastewater (Table 1).

**Table 1:** Heavy metals concentration in Tannery's wastewater.

**Heavy metals**	**10%** **(mgL^−1^)**	**20%** **(mgL^−1^)**	**30%** **(mgL^−1^)**	**50%** **(mgL^−1^)**	**75%** **(mgL^−1^)**	**100%** **(mgL^−1^)**
Cr	132	219	291	407	489	923
Pb	1.879	2.867	4.795	5.672	7.245	10.547
Cu	0.138	0.145	0.166	0.204	0.235	0.278
Ni	0.052	0.197	0.322	0.815	1.214	1.627
Mn	0.037	0.133	0.233	0.520	0.709	0.872
Cd	0.036	0.066	0.095	0.136	0.194	0.234

### Preparation of bacterial inoculum, inorganic, and organic amendments

Pure cultures of bacterial strains *Trichococcus* sp. (FCBP-SB-0254), *Pseudomonas alcaligenes* (FCBP-SB-0372), and *Bacillus subtilis* (FCBP-SB-0189) (B1, B2, and B3, respectively) were selected through screening based on *in vitro* laboratory trial and these were acquired from First Fungal Culture Bank of Pakistan (FCBP) at the Faculty of Agricultural Sciences, University of the Punjab, Lahore. Bacterial strains (B1, B2, and B3) were cultured in Luria-Bertani (LB) broth at 37°C for 48 h to get more biomass in the broth (however, bacterial cultures can be fully prepared within 24 h) (Loutfi et al., 2020).

Pure silica gel polymerized with chitosan (CP-silica gel) was mixed at the rate of 0.2% [as CP-silica gel is highly porous in structure, so its minimum quantity can efficiently adsorb a considerable number of heavy metals (Omura et al., 2021)] in 1 kg of soil for pot experiment. Brunauer–Emmett–Teller (BET) analysis were performed on the NovaWin 20e (Quanta Chrome, Virginia, United States) instrument (which used nitrogen gas for analysis) to analyze the morphological features [Physical structure (Inorganic, Silica gel beads), Pore size (111.89 nm), BET surface area (104.4 m^2^/g), Pore volume, P/Po (0.123 cm^3^/g), Micropore volume (0.00203 cm^3^/g), and Density (1.000 g/cm^3^) (Dutta et al., 2005)] of CP-silica gel. Zinc-doped biochar (ZnBc) was prepared at the rate of 30% Zn(NO_3_)_2_. 6H_2_O added into 10 g of rice husk biochar (RHBc) (Hossain et al., 2020) (pyrolysis was done in a Furnace at 500°C temperature because RHBc exhibits a large degree of porosity with contents of oxygen, potassium, and carbon at a 1-10-micron scale) (Van Vinh et al., 2015); distilled water (d. H_2_O) was added with continuous stirring at hot plate for 2 h. The temperature was maintained by giving continuous heat to the vessel at 80–100°C. The process was completed after one and half hours through filtration assembly; then, ZnBc was filtered. In the end, to get the desired enriched biochar, the residue was dried in the air following oven drying for 24 h (Alexander et al., 2017). Finally, 1.5% ZnBc [simple biochar performs more efficiently to adsorb heavy metals when enriched with zinc and other minerals (Liu et al., 2021)] was used in the pot experiment and characterized [Moisture content (0.08%), pH (5.64), Electrical conductivity (0.025 dS/m), Bulk density (0.404 g/cm^3^), Particle density (0.614 g/cm^3^), Pore space (34.20%), Cation exchange capacity (31.18 cmol kg^−1^), and Zinc content (82.034 mg L^−1^) (Yang et al., 2018)] in the laboratory.

#### Morphological characteristics and biosorption trial of bacterial isolates (study I)

Bacterial strains were cultured on nutrient agar (NA) media (Treguier et al., 2021) (to culture the strains in solid form on a solid medium) for further characterization. First, 2.2% NA and 1% NaCl was dissolved in 500 ml of deionized water. The solution pH was also adjusted up to pH 7 with the addition of NaOH. The solution of NA media was autoclaved for 20 min at 15 psi, cooled up to 55°C, and poured into Petri dishes (Loutfi et al., 2020). Then, reinoculation was done by following the method given by Sun et al. (2018) to purify bacterial isolates. The characterization of bacterial isolates (gram, shape, and color tests) was done through Gram stain by following the method of Zhang et al. (2015). For the viability of bacterial species to produce spores, spore staining was done by following the method given by Hahne et al. (2019) and spores were observed under an oil emersion lens of a microscope (OLYMPUS-CX23). The bacterial isolates were also examined under ultraviolet light (UV test). For this purpose, streaks of bacterial isolates (B1, B2, and B3) were analyzed under Electronic Dual Light-Transilluminator (PATENT-543446) (Andrey et al., 2019). Then, the photographs of prepared slides and Petri dishes (gram and spore staining) were taken with a digital camera (HD 1500T Meiji Techno Japan).

Biosorption of total Cr and Pb was investigated by inoculating pure bacterial isolates (B1, B2, and B3) into total Cr and Pb (30% wastewater formulation) (Table 1) containing the broth. A total of 1 ml of bacterial cultures (overnight grown on wastewater) was carefully inoculated into 100 ml of sterilized broth separately in round bottom flasks (250 ml). The flasks were incubated at 30°C for 24 h. After incubation, the bacterial cultures were centrifuged at 13,000 rpm for 10 min. The supernatants were collected to check the residual amount of total Cr and Pb through an atomic absorption spectrophotometer (AAS) (Arif et al., 2019).

### Pot experiment (study II)

This study was conducted in the greenhouse at Botanic Garden, Government College University Lahore located at 31°33′41.94"E and 74°19′41.94"N. The average maximum and minimum temperatures were 40 and 27°C, respectively, and the temperature of the research site was 28–32°C. There were 12 treatments such as T1 = Control; T2 = CP-silica gel (0.2%); T3 = ZnBc (1.5%); T4 = B1; T5 = B2; T6 = B3; T7 = CP-silica gel (0.2%) + B1; T8 = CP-silica gel (0.2%) + B2; T9 = CP-silica gel (0.2%) + B3; T10 = ZnBc (1.5%) + B1; T11 = ZnBc (1.5%) + B2 and T12 = ZnBc (1.5%) + B3 applied in the pots having 3 kg soil. The physicochemical properties [Soil texture (Clay loam), Sand (38%), Silt (32%), Clay (36%), Organic matter (0.86 %), pH (7.48), EC (1.37 dSm^−1^), CEC (5.96 cmol Kg^−1^), DTPA-Cr (0.2018 mg Kg^−1^) and DTPA-Pb (0.6873 mg Kg^−1^) (Robertson et al., 1999)] of the bhal soil used in this experiment were analyzed in the laboratory. Each treatment was replicated three times and an adequate amount of wastewater was applied to all pots to maintain soil field capacity. Then, brinjal (*Solanum melongena* L.) was selected for the pot experiment as it is the most widely used vegetable crop grown due to the high availability of nutrition, antioxidants, and vitamins. For this purpose, 7 days old seedlings of *Solanum melongena* L. were transplanted into each pot. After 2 days of transplantation, the recommended dose of fertilizer such as urea and diammonium phosphate (DAP) was added to the pots.

After 15 days of transplantation, the plants were harvested; the roots and shoots of plants were carefully extracted and washed with distilled water to remove the debris materials. The plants and soil samples were stored in labeled zipper bags and carried in the laboratory for further analysis.

### Measurements and analysis

#### pH and EC (electrical conductivity) of soil

In a 100 ml beaker, 50 g of soil sample was taken and 50 ml of distilled water was added. The sample was thoroughly mixed, and 30 min were given to stand. After 10 min, the suspension was stirred well. Then, after 1 h, the suspension was stirred again and the pH of the soil was measured by using a pH meter (YSI pH100) (Hailegnaw et al., 2019).

A total of 50 g of sample soil was oven dried for 2 h and taken in a 100 ml beaker. Then 50 ml volume of distilled water was added in the beaker and mixed thoroughly. After a wait of 30 min, the suspension was stirred for another 10 min. Finally, after 1 h, the suspension was again stirred and filtered through Whatman filter paper. Then, the EC of the clear soil solution was checked by using an EC meter (Extech EC300) (Hailegnaw et al., 2019).

#### Fresh weight and dry weight of roots, stems, and leaves and Chl. content of Solanum melongena L. plant

After harvesting, fresh weight of roots and shoots of all the replicates were immediately measured by using a standard weighing balance. Plants were dried in the open air for 2 days and then in a hot oven at 65°C for 24 h and measured separately (roots, stems, and leaves) by using a standard weighing balance.

By following the method of Du et al. (2017), chlorophyll content was measured in freshly harvested leaves of *Solanum melongena* L. plant. The sample extract was prepared by taking 1 g of leaf and ground in 90% acetone by using a pestle and mortar and absorbance was measured at 663 and 645 nm wavelengths by using a UV visible spectrophotometer (Spectro scan 80D). Chlorophyll a and b contents were measured by following these equations:


(1) $$\begin{array}{r} Chl.\;a\;\left(mg\;ml-1\right)=\;\left[11.64\;\times \;\left(A663\right)\;2.16\;\times \;\left(A645\right)\right] \end{array}$$

(2) $$\begin{array}{r} Chl.\;b\;\left(mg\;ml-1\right)=\;\left[20.97\;\times \;\left(A645\right)\;3.94\;\times \;\left(A663\right)\right] \end{array}$$

However, (A663) and (A645) are the absorbance values detected at the wavelength of 645 and 663 nm, respectively.

#### Heavy metal analysis for soil and plants

The concentrations of heavy metals such as total Cr and Pb in soil were determined by the method of DTPA (diethylene triamine penta acetic acid) extraction. DTPA extraction was carried out by taking 1.97 g of DTPA and 1.1 g of CaCl_2_ (calcium chloride) in a beaker and transferring them to a 1L volume d. H_2_O. Then, 14.92 g of TEA (triethanolamine) was added into another beaker and then transferred to a 1 L flask and brought to the volume of about 900 ml by adding d. H_2_O. By using 6N HCl (hydrochloric acid), the pH was adjusted up to neutral and brought the final 1 L volume. This solution had 0.005 M of DTPA and 0.1 M of TEA and CaCl_2_ (Xiao et al., 2020). Similarly, the concentrations of total Cr and Pb in roots, stems, and leaves of *Solanum melongena* L. plant were determined by the method of acid digestion. For acid digestion, HNO_3_+HClO_4_ (2:1) was used to digest plants for the analysis of total Cr and Pb. Standards were prepared for the analysis of heavy metals on a multi-sequential AAS (Golui et al., 2020).

#### Biochemical analysis of ROS in Solanum melongena L. plant

To measure the concentration of H_2_O_2_ (Abdel Latef et al., 2019), sample extract of fresh root, stem, and leaf was prepared by homogenizing 500 mg tissue with 5 ml of TCA (0.1%) solution and centrifuged for 15 min at 12,000 rpm. Then, 1 ml of supernatant was taken and 1 ml of 10 mM K-P buffer with pH 7.0 and 2 ml of 1 M potassium iodide (KI) solution were added to it. Absorbance was calculated at 390 nm wavelength by using a spectrophotometer and the concentration of H_2_O_2_ was expressed in μmol g^−1^.

To measure the concentration of MDA (malondialdehyde), 5 ml of 20% TCA solution (trichloroacetic acid) was used to ground 0.5 g of fresh *Solanum melongena* L. leaves and the sample mixture was centrifuged at 10,000 rpm for 10–15 min. The supernatant (2.5 ml) was separated in a test tube and 20% (v/w) TCA and 0.5% (v/w) TBA (thiobarbituric acid) solutions were added with a volume of 1 ml in it. In a dry oven, the mixture was heated for 30 min at 95°C and then cooled at freezing temperature. A spectrophotometer was used to measure the absorbance of the mixture at 532 nm and 600 nm wavelengths and by using Beer and Lambert's equation, the concentration of MDA in μmol g^−1^ was calculated (Kumar and Pathak, 2018).

According to Zhao et al. (2020), CMP (cell membrane permeability) was measured by taking 1 g of fresh *Solanum melongena* L. leaves dipped into d. H_2_O (20 ml) and placed for 12 h in the shaker. Then, by using EC (electrical conductivity) meter, EC1 was measured. After this, the mixture was autoclaved at 121°C for 25 to 30 min and EC2 measured after cooling at room temperature (Zhao et al., 2020). Finally, the percentage of CMP was calculated by using the formula:


(3) $$\begin{array}{r} CMP=EC1/EC2\;\times 100 \end{array}$$

#### Antioxidant enzyme assays

To prepare sample extract, 0.2 g of fresh root, stem, and leaf was homogenized in 1.2 ml of 0.2 M K-P buffer with pH 7.8 and centrifuged at 15,000 rpm at 4°C for 20 min. The supernatant was separated and the pellet was collected and resuspended in the same buffer with 0.8 ml volume. The suspension was again centrifuged at 15,000 rpm for 15 min. The supernatant was combined and stored at freezing temperature and used to measure different activities of antioxidant enzymes (Jardim-Messeder et al., 2018).

A total of 1 ml extract was taken and diluted with 50 mM K-P buffer (pH 7) 200 times. Then, 2 ml of that mixture was taken and 1 ml of H_2_O_2_ (10 mM) was added into it. Absorbance was recorded through a spectrophotometer at 240 nm wavelength to express the activity of CAT (catalase) in μmol g^−1^ (Jardim-Messeder et al., 2018).

The sample extract (40 μl) was taken and mixed with 1,320 μl of 50 mM K-P buffer (pH 7.0), 1,320 μl of 0.5 mM Ascorbate, and 1,320 μl of 0.5 mM H_2_O_2_. Absorbance was recorded through a spectrophotometer at 290 nm wavelength to express the activity of APX (ascorbate peroxidase) in μmol g^−1^ (Jardim-Messeder et al., 2018).

#### Secondary metabolites analysis in plants

A method by Metwally et al. (2021) was used to determine the concentrations of protein in roots, stems, and leaves of *Solanum melongena* L. plant. A total of 0.1 g of root or shoot samples were taken and 4 ml of 80% acetone was used to ground samples to prepare the sample extract. Then, in a test tube, sample extract with 200 μl volume was mixed with 1,800 μl d. H_2_O. Then, 2 ml of Bradford reagent was added to the mixture and placed for 10–20 min at room temperature for incubation. A spectrophotometer was used to measure the absorbance at 595 nm after incubation and standard curve was obtained through various concentrations of BSA (Bovine Serum Albumin).

By following Ahmed et al.'s (2019) method, total phenolics contents were measured in the root, stem, and leaf of the *Solanum melongena* L. plant. For this purpose, 20 μl of sample extract was taken in a test tube and 100 μl of Folin–Coicâlteu's reagent (0.25 N), 1,580 μl of d. H_2_O, and 300 μl of Na_2_CO_3_ solution were added to it. A time of 2 h was given to the mixture to remain in a dark at room temperature, and finally, at 760 nm wavelength, absorbance and gallic acid standards of the mixture were measured by using a spectrophotometer. The concentration of total phenolics was represented in μg g^−1^.

#### Bioaccumulation, translocation factors, and percent variance analysis

Bioconcentration factor (BCF) and Translocation factor (TF) of heavy metals in *Solanum melongena* L. plant were determined by following these equations (Takarina and Pin, 2017):


(4) $$\begin{array}{rcl} BCF & = & \frac{\text{Conc}.\text{ of heavy metal in shoot}/\text{root}}{\text{Conc}.\text{ of heavy metal in soil}} \end{array}$$

(5) $$\begin{array}{rcl} TF & = & \frac{\text{BCF in stem}}{\text{BCF in root}} \end{array}$$

Percent variance analysis of soil was determined to check the efficiency of treatments applied in this research study to remove heavy metal stress in soil. It was carried out by following this equation (Takarina and Pin, 2017):


(6) $$\begin{array}{r} \text{Percent Variance Analysis }\left(\text{\%}\right)=\frac{\left(\text{Conc}.\text{ of heavy metal in control Conc}.\text{ of heavy metal in treatments}\right)}{\text{Conc}.\text{ of heavy metal in control}} \end{array}$$

### Statistical analysis

Data were statistically analyzed by Microsoft Excel 2019^®^ and Statistix 8.1^®^ (Analytical Software, Tallahassee, United States) was used to design one-way ANOVA (analysis of variance) of the above-mentioned treatments. The Least Significant Difference (LSD) test was applied to the dataset to compare the means of groups through Statistix 8.1^®^ (Dasgupta, 2019). Principal Component Analysis (PCA-Pearson correlation) was performed by using XLSTAT version 2021 (Farhangi-Abriz and Ghassemi-Golezani, 2021). For this purpose, active variables were selected to evaluate the best results among the parameters of soil, roots, stems, and leaves and the main components were selected as F1 and F2 because these showed significant contributions to generating biplots. Sigmaplot 14.0 was used for the graphical representation of the results (Ishfaq et al., 2021). All the results were the mean of three replicates (*n* = 3) with standard error (SE).

## Results

### Morphological characterization and biosorption traits of bacterial sp.

**Figure 1:**
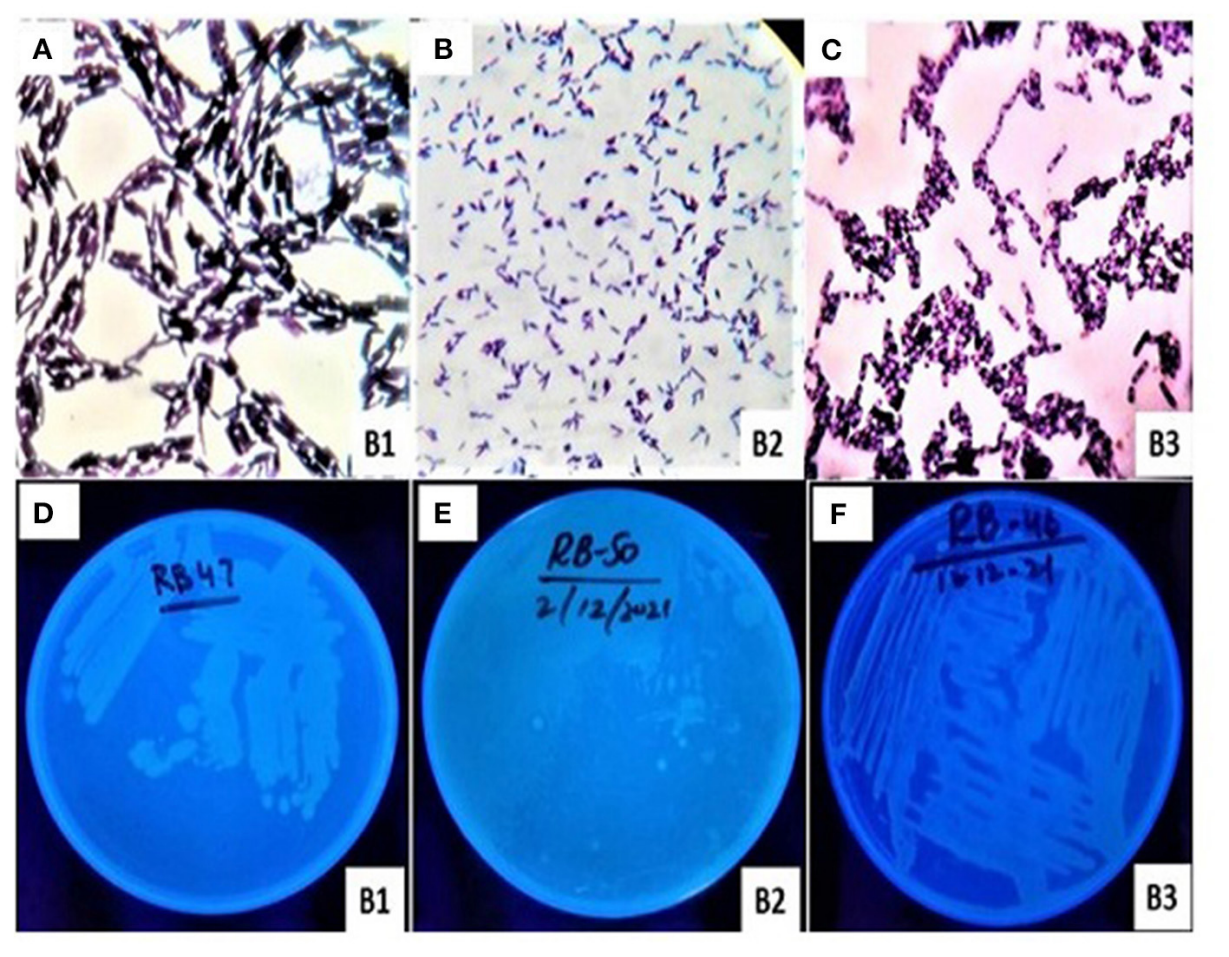
Morphological characterization of bacterial isolates (B1, B2, and B3) in **(A–C)** and photographs under ultraviolet radiations in **(D–F)**, respectively.

The morphological characteristics such as shape, color, gram test, UV test, and spore test of bacterial sp. (B1, B2, and B3) were cautiously investigated (Table 2; Figure 1). These bacterial isolates were rod shaped and had shown different characters in gram and spore testing (Figure 1; Table 2). B1 and B2 were significantly similar (gram-positive) to each other in gram testing, while B3 was investigated as gram-negative. However, B2 and B3 were investigated as significantly positive in spore testing, while B1 was examined as negative in spore testing. The bacterial isolates also showed significant dissimilarities in color. These were off-white except B1 while under UV (ultraviolet) radiations isolates were bright except B2 (Figure 1).

**Table 2:** Morphological and biosorption characteristics of bacterial isolates.

**Characteristics**	**B1**	**B2**	**B3**
**Name**	***Trichococcus*** **sp**.	***Pseudomonas***	***Bacillus***
		***alcaligenes***	***subtilis***
Shape	Rod	Rod	Rod
Gram test	-	-	+
Spore test	-	+	+
UV test	Bright	Dull	Bright
Color	Yellow	Off-white	Off-white
Cr (mg L^−1^)	249.24	234.56	232.79
Pb (mg L^−1^)	0.2367	0.3785	0.1578

*The signs “–” and “+” stands for “negative” and “positive”*.

All bacterial strains demonstrated relatively high total Cr and Pb resistance. Out of three heavy metals resistant isolates, B3 showed maximum total Cr removal efficiency up to 6 and 1% when compared with B1 and B2, respectively (Table 2). Similarly, biosorption of total Pb also has been efficiently increased by bacterial isolates. It was investigated that B3 showed the most effective sorption (33 and 58%) of total Pb when compared with B1 and B2, respectively (Table 2).

### Scenario of physiological attributes and stress tolerance

Wastewater application significantly (*p* ≤ 0.01) reduced the plant's fresh weight (FW), dry weight (DW), and chlorophyll content relative to all other treatments (Table 3). However, CP-silica gel, ZnBc, and bacterial sp. significantly (*p* ≤ 0.01) enhanced the growth rate of the *Solanum melongena* L. plant (Table 3). The application of CP-silica gel + B3 proved to be an effective treatment which increased (3-folds) FW in leaf when compared to control while DW most effectively increased (12-folds) in the leaf under ZnBc + B3 when compared to control.

**Table 3:** Fresh and dry weight of root, stem, and leaf, and leaf chlorophyll content of *Solanum melongena* L. plant determined after harvesting under different treatments against heavy metal stress.

**Coding**	**Treatments**	**Root**		**Stem**		**Leaf**	
		**F.W (g)**	**D.W (g)**	**F.W (g)**	**D.W (g)**	**F.W (g)**	**D.W (g)**	**Chl. a**	**Chl. b**
T1	Control + W.W	0.15 ± 0.02i	0.01 ± 0.005c	0.86 ± 0.15f	0.04 ± 0.005i	0.55 ± 0.17g	0.02 ± 0.005g	5.40 ± 0.31f	2.53 ± 0.12f
T2	CP-Si gel + W.W	0.36 ± 0.01f	0.04± 0.005bc	1.53 ±0.10cde	0.15 ± 0.01fg	1.18 ± 0.15f	0.12 ± 0.01e	8.21 ± 0.29d	3.06 ± 0.09e
T3	ZnBc + W.W	0.39 ± 0.01e	0.06 ± 0.005ab	1.71 ± 0.15c	0.17 ± 0.005ef	1.23 ± 0.05ef	0.14 ± 0.005de	5.76 ± 0.57 f	3.97 ± 0.15c
T4	B1 + W.W	0.26 ± 0.01h	0.04 ± 0.002bc	1.35 ± 0.15de	0.12 ± 0.01g	1.34 ± 0.05ef	0.13 ± 0.01de	7.27 ± 0.29e	2.98 ± 0.09ef
T5	B2 + W.W	0.25 ± 0.01h	0.04 ± 0.005bc	1.23 ± 0.11e	0.08 ± 0.005h	0.89 ± 0.11g	0.06 ± 0.01f	8.15 ± 0.22d	3.92 ± 0.11c
T6	B3 + W.W	0.32 ± 0.02g	0.07 ± 0.007ab	1.60 ± 0.09 cd	0.16 ± 0.01ef	1.41 ± 0.12def	0.15 ± 0.01cde	8.52 ± 0.33cd	3.98 ± 0.24c
T7	CP-Si gel+B1+W.W	0.36 ± 0.02ef	0.05± 0.005bc	1.78 ± 0.16c	0.19 ± 0.01de	1.48 ± 0.10de	0.13 ± 0.01de	8.51 ± 0.19cd	3.26 ± 0.07d
T8	CP-Si gel+B2+W.W	0.43 ± 0.01d	0.06 ± 0.003ab	1.88 ± 0.18bc	0.18 ± 0.01def	1.60 ± 0.05cd	0.16 ± 0.01bcd	10.4 ± 0.06b	4.09 ± 0.11bc
T9	CP-Si gel+B3+W.W	0.54 ± 0.02b	0.07 ± 0.006ab	1.73 ± 0.13c	0.21 ± 0.01cd	1.81 ± 0.15bc	0.18 ± 0.01bc	10.8 ± 0.37ab	4.69 ± 0.22a
T10	ZnBc+B1+W.W	0.45 ± 0.01d	0.06 ± 0.005ab	1.71 ± 0.06c	0.23 ± 0.01c	1.32 ± 0.11ef	0.15 ± 0.01cde	8.10 ± 0.18d	4.10 ± 0.31bc
T11	ZnBc+B2+W.W	0.48 ± 0.02c	0.06 ± 0.003ab	2.18 ± 0.06b	0.44 ± 0.01b	1.89 ± 0.11b	0.19 ± 0.01b	8.79 ± 0.15c	4.38 ± 0.12ab
T12	ZnBc+B3+W.W	0.76 ± 0.01a	0.08 ± 0.006a	2.76 ± 0.15a	0.56 ± 0.01a	2.98 ± 0.16a	0.27 ± 0.01a	10.7 ± 0.12a	4.56 ± 0.09a

*Data in Table 3 represents the mean values of three replicates (n = 3) ± standard error and different letters represent significant differences declared by the LSD test at p ≤ 0.05*.

*W.W-Wastewater; CP-Si gel-Chitosan polymerized silica gel; ZnBc-Zinc doped biochar; B1-Trichoccocuss sp.; B2-Psuedomonas alcaligenes; B3-Bacillus subtilis; F.W-Fresh weight; D.W-Dry weight; Chl. a-Chlorophyll a; Chl. b-Chlorophyll b*.

The application of wastewater significantly (*p* ≤ 0.01) decreased the chlorophyll content in the *Solanum melongena* L. plant when compared to all other treatments (Table 3). Chlorophyll a revealed that CP-silica gel + B3 performed better (one-fold) when compared to control; similarly, chlorophyll b also significantly increased (86%) in the plant under CP-silica gel + B3. Interestingly, B3 doubled the effectiveness of CP-silica gel and ZnBc.

**Figure 2:**
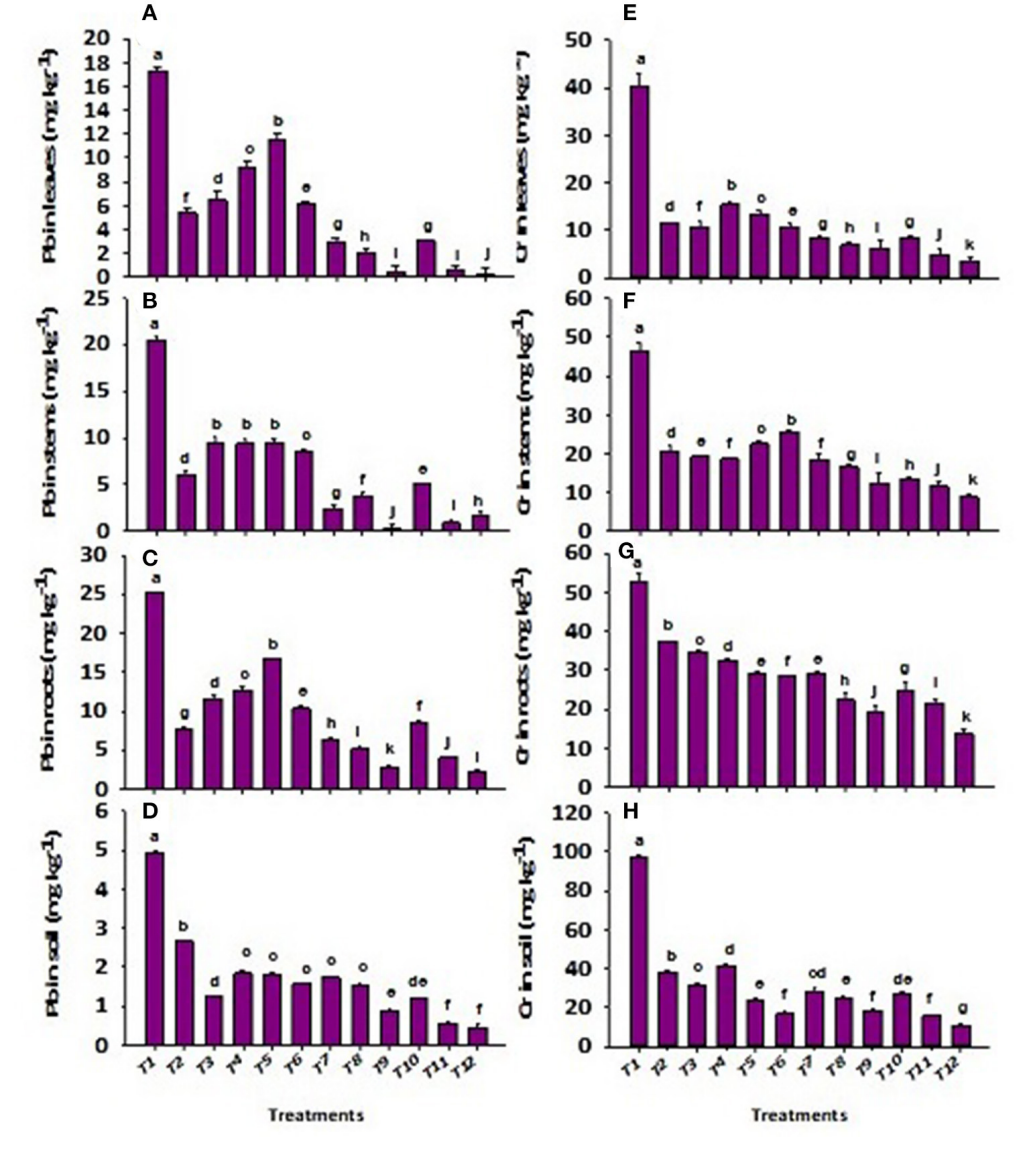
Concentration of total lead (Pb) in **(A)** leaf, **(B)** stem, **(C)** root, **(D)** soil and chromium (Cr) in **(E)** leaf, **(F)** stem, **(G)** root and **(H)** soil of *Solanum melongena* L. plant grown in heavy metal contaminated soil (wastewater applied as stress) under different treatments such as T1 = Control; T2 = CP-Silica gel; T3 = ZnBc; T4 = B1; T5 = B2; T6 = B3; T7 = CP-Silica gel + B1; T8 = CP-Silica gel + B2; T9 = CP-Silica gel + B3; T10 = ZnBc + B1; T11 = ZnBc + B2 and T12 = ZnBc + B3. Values are the average of three replicates ± standard error (SE). Mean bars sharing similar lower case alphabetic letters were non-significant (*p* < 0.05) to each other while different letters represented significant difference declared by one-way ANOVA using LSD test at *p* ≤ 0.05.

Application of wastewater significantly (*p* ≤ 0.01) increased the concentrations of total Cr and Pb in soil and plant (Figure 2). However, the effect of total Cr and Pb stress was significantly (*p* ≤ 0.01) regulated in soil under CP-silica gel, ZnBc, and bacterial sp. (Figure 2). The effect of CP-silica gel + B3 was investigated as an efficient stress-tolerant which significantly decreased the concentrations of total Cr (81%) and Pb (82%) in soil; however, ZnBc + B3 proved to be the most effective treatment to regulate total Cr (89%) and Pb (91%) stress in tanneries affected soil. Similarly, total Cr and Pb stress were also significantly (*p* ≤ 0.01) regulated by the applications of CP-Silica gel, ZnBc, and bacterial sp. (especially B1 and B3) (Figure 2). CP-Silica gel + B3 proved as an effective treatment which significantly decreased the concentration of total Cr (84%) and Pb (97%) in the leaf when compared to control. While ZnBc + B3 proved as a most effective treatment to reduce the concentrations of total Cr (92%) and Pb (98%) in the leaf as compared to control.

### Scenario of oxidative damage and antioxidant defense system

Wastewater application significantly (*p* ≤ 0.01) enhanced the H_2_O_2_ and MDA concentrations in all parts of the plant which further led to the overproduction of CMP (Table 4). However, bacterial sp. in combination with CP-Silica gel and ZnBc significantly (*p* ≤ 0.01) reduced the H_2_O_2_ and MDA compared to control (Table 4). The application of CP-Silica gel + B3 proved to be an effective treatment to minimize (83%) the stem MDA contents when compared to control; however, maximum reduction (91%) in stem H_2_O_2_ was observed with ZnBc + B3. Although, the main effects and first-order interaction were significantly influenced by the bacterial sp., CP-Silica gel, and ZnBc; however, ZnBc + B3 proved as the most effective treatment to regulate (72%) the CMP in the leaf relative to control.

**Table 4:** Hydrogen peroxide (H_2_O_2_), malondialdehyde (MDA) and cell membrane permeability (CMP) of root, stem, and leaf of *Solanum melongena* L. plant were determined under different treatments against wastewater stress.

**Coding**	**Treatments**	**Root**			**Stem**			**Leaf**		
		**H_2_O_2_** **(μmol g^−1^)**	**MDA** **(μmol** **g^−1^)**	**CMP (%)**	**H_2_O_2_** **(μmol g^−1^)**	**MDA** **(μmol g^−1^)**	**CMP (%)**	**H_2_O_2_** **(μmol g^−1^)**	**MDA (μmol** **g^−1^)**	**CMP (%)**
T1	Control + W.W	0.26 ± 0.01a	0.72 ± 0.02a	82.4 ± 1.01a	0.24 ± 0.01a	0.58 ± 0.02a	104 ± 1.07a	0.36 ± 0.01a	3.25 ± 0.02a	233 ± 1.51a
T2	CP-Si gel + W.W	0.18 ± 0.01b	0.55 ± 0.01b	63.5 ± 1.42e	0.12 ± 0.01cd	0.34 ± 0.01c	86.3 ± 0.65c	0.22 ± 0.01c	1.39 ± 0.04d	160 ± 1.55c
T3	ZnBc + W.W	0.16 ± 0.01b	0.34 ± 0.03e	67.9 ± 0.53d	0.18 ± 0.01b	0.45 ± 0.01b	94.7 ± 0.57b	0.24 ± 0.01c	1.55 ± 0.03c	178 ± 0.73b
T4	B1 + W.W	0.05 ± 0.01def	0.31 ± 0.02e	74.7 ± 1.48b	0.15 ± 0.01bc	0.45 ± 0.01b	95.5 ± 1.12b	0.28 ± 0.01b	1.91 ± 0.07b	154 ± 1.56d
T5	B2 + W.W	0.03 ± 0.01f	0.26 ± 0.01f	71.5 ± 1.06c	0.09 ± 0.01de	0.25 ± 0.01d	86.4 ± 0.82c	0.22 ± 0.01c	1.82 ± 0.05b	132 ± 2.09f
T6	B3 + W.W	0.02 ± 0.01f	0.22 ± 0.02g	69.5 ± 0.91cd	0.06 ± 0.01efg	0.22 ± 0.01de	82.4 ± 0.52d	0.15 ± 0.01e	1.16 ± 0.06e	134 ± 1.15f
T7	CP-Si gel+B1+W.W	0.06 ± 0.01de	0.48 ± 0.01c	62.8 ± 1.61e	0.06 ± 0.01efg	0.21 ± 0.02ef	81.6 ± 0.84d	0.12 ± 0.01ef	1.04 ± 0.03e	143 ± 0.98e
T8	CP-Si gel+B2+W.W	0.12 ± 0.01c	0.52 ± 0.02b	45.9 ± 1.49g	0.05 ± 0.01fgh	0.14 ± 0.02ghi	68.3 ± 1.04e	0.18 ± 0.01d	0.86 ± 0.03f	93.4 ± 1.25h
T9	CP-Si gel+B3+W.W	0.04 ± 0.01ef	0.41 ± 0.03d	59.2 ± 0.65f	0.03 ± 0.01gh	0.11 ± 0.03i	52.8 ± 1.12g	0.09 ± 0.01gh	0.65 ± 0.03g	90.5 ± 1.07h
T10	ZnBc+B1+W.W	0.07 ± 0.01d	0.21 ± 0.02g	58.2 ± 0.63 f	0.08 ± 0.01ef	0.17 ± 0.01fg	89.1 ± 1.26c	0.15 ± 0.01e	1.14 ± 0.04e	122 ± 0.89g
T11	ZnBc+B2+W.W	0.06 ± 0.01de	0.18 ± 0.01g	47.5 ± 2.16g	0.05 ± 0.01fgh	0.15 ± 0.01gh	61.6 ± 1.08f	0.11 ± 0.01fg	0.74 ± 0.03fg	83.4 ± 1.96i
T12	ZnBc+B3+W.W	0.03 ± 0.01f	0.08 ± 0.02h	38.1 ± 0.98h	0.02 ± 0.01h	0.12 ± 0.02hi	49.4 ± 1.01h	0.07 ± 0.01h	0.63 ± 0.03g	65.6 ± 1.48j

*Data represented the mean value of three replicates (n = 3) ± standard error and different letters represent significant differences declared by the LSD test at p ≤ 0.05*.

*W.W-Wastewater; CP-Si gel-Chitosan polymerized silica gel; ZnBc-Zinc doped biochar; B1-Trichoccocuss sp; B2-Psuedomonas alcaligenes; B3-Bacillus subtilis*.

**Figure 3:**
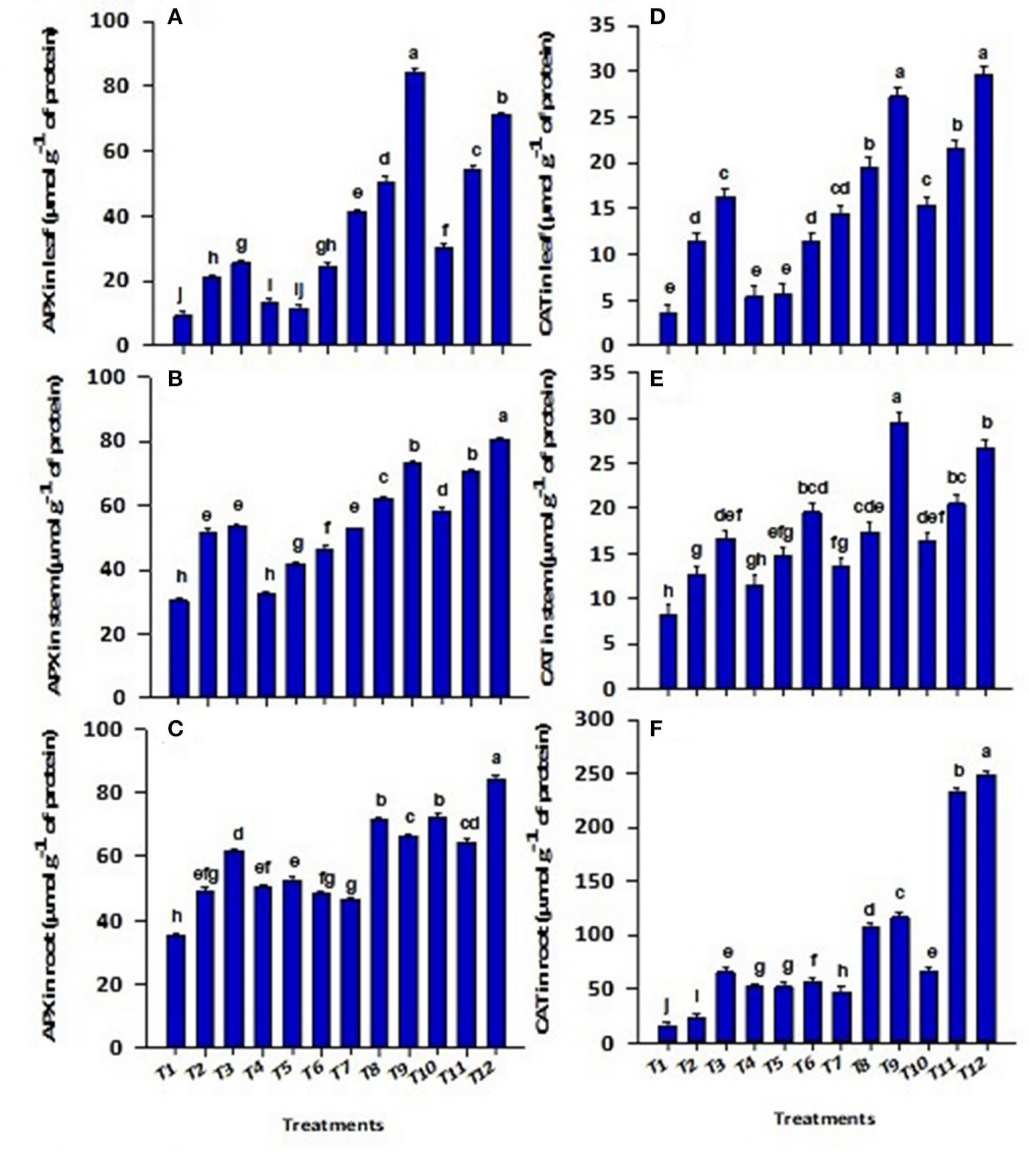
Concentration of ascorbate peroxidase (APX) in **(A)** leaf, **(B)** stem, **(C)** root and catalase (CAT) in **(D)** leaf, **(E)** stem and **(F)** root of *Solanum melongena* L. plant grown in heavy metal contaminated soil (waste water applied as stress) under different treatments such as T1 = Control; T2 = CP-Silica gel; T3 = ZnBc; T4 = B1; T5 = B2; T6 = B3; T7 = CP-Silica gel + B1; T8 = CP-Silica gel + B2; T9 = CP-Silica gel + B3; T10 = ZnBc + B1; T11 = ZnBc + B2 and T12 = ZnBc + B3. Values are the average of three replicates ± standard error (SE). Mean bars sharing similar lower case alphabetic letters were non-significant (*p* < 0.05) to each other while different letters represent significant difference declared by one way ANOVA using LSD test at *p* ≤ 0.05.

Similarly, APX and CAT activities were significantly (*p* ≤ 0.01) influenced by the first-order interactions of bacterial sp., CP-Silica gel, and ZnBc (Figure 3). Wastewater application significantly (*p* ≤ 0.01) reduced the APX and CAT activities irrespective of the plant part and maximum reduction in leaf CAT (89%) was reported in control relative to ZnBc + B3. Application of bacterial sp. enhanced the APX and CAT contents but the effect of B3 was well pronounced when combined with CP-Silica gel and ZnBc. The application of CP-Silica gel + B3 induced (six-folds) the activities of APX in the leaf when compared to control; however, a maximum increase (15-folds) in CAT was observed in the root amended with ZnBc + B3.

### Scenario of secondary metabolites to combat oxidative damage

**Figure 4:**
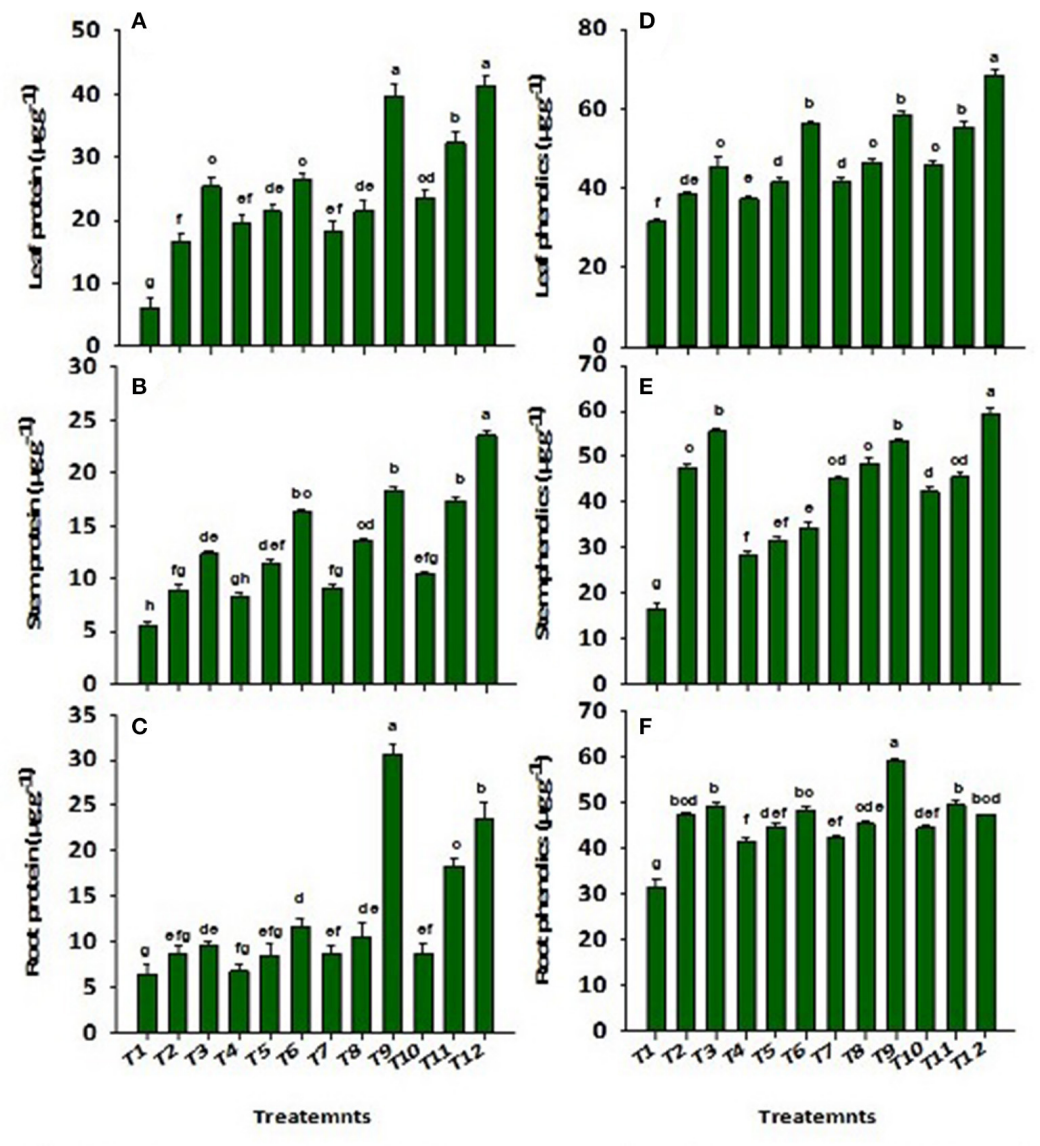
Concentration of proteins in **(A)** leaf, **(B)** stem, **(C)** root and phenolics in **(D)** leaf, **(E)** stem and **(F)** root of *Solanum melongena* L. plant grown in heavy metal contaminated soil (waste water applied as stress) under different treatments such as T1 = Control; T2 = CP-Silica gel; T3 = ZnBc; T4 = B1; T5 = B2; T6 = B3; T7 = CP-Silica gel + B1; T8 = CP-Silica gel + B2; T9 = CP-Silica gel + B3; T10 = ZnBc + B1; T11 = ZnBc + B2 and T12 = ZnBc + B3. Values are the average of three replicates ± standard error (SE). Mean bars sharing similar lower case alphabetic letters were non-significant (*p* < 0.05) to each other while different letters represent significant difference declared by one way ANOVA using LSD test at *p* ≤ 0.05.

Wastewater application significantly (*p* ≤ 0.01) reduced the concentrations of proteins and total phenolics in the root, stem, and leaf of *Solanum melongena* L. (Figure 4). There was a decrease (86%) and (54%) in leaf proteins and phenolics, respectively, reported in control as compared to ZnBc + B3. However, the application of CP-Silica gel, ZnBc, and bacterial sp. (B1, B2, and B3) significantly (*p* ≤ 0.01) enhanced the proteins and total phenolics in the *Solanum melongena* L. plant when compared to control. Similarly, CP-Silica gel and ZnBc showed better performance when combined with B3. The results of proteins were more pronounced (six-folds) in the leaf by the application of CP-Silica gel + B3 and ZnBc + B3 when compared to control while total phenolics were most effectively increased (three-folds) in the stem when treated with ZnBc + B3.

### Principal component analysis

**Figure 5:**
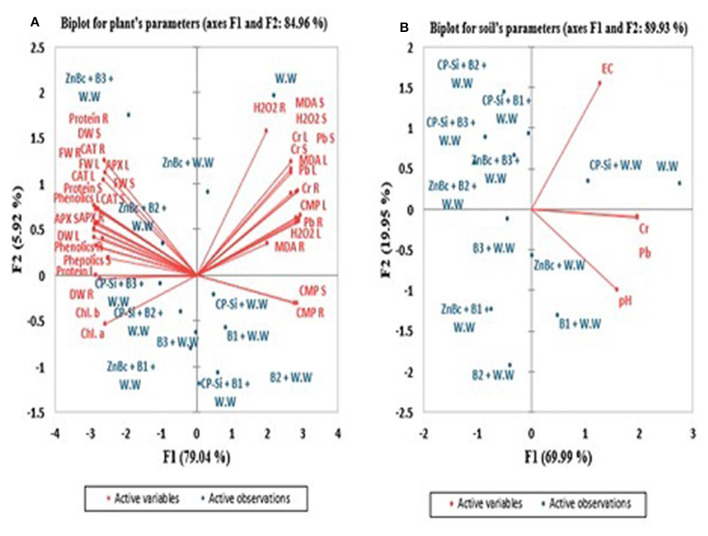
Principal component analysis (PCA) of *Solanum melongena* L. plant and soil. F1 and F2 are PCA main factors/components which significantly contributed to generate PCA biplots. Active variables are **(A)** Chlorophyl a (Chl. a), Chlorophyl b (Chl. b), total Chromium (Cr), total Lead (Pb), Hydrogen peroxide (H_2_O_2_), Malondialdehyde (MDA), Cell membrane permeability (CMP), Ascorbate peroxidase (APX), Catalase (CAT), proteins and phenolics and **(B)** parameters of soil such as total Cr and Pb, pH and Electrical conductivity (EC); however, R, S and L stands for root, stem and leaf respectively. Active observations are W.W-Wastewater; CP-Si gel-Chitosan polymerized silica gel; ZnBc-Zinc doped biochar; B1-Trichoccocuss sp; B2-Psuedomonas alcaligenes; B3-Bacillus subtilis.

Physiological and biochemical parameters of *Solanum melongena* L. plant and soil characters were analyzed by constructing biplots of PCA to evaluate the most efficient treatment used in the proposed study (Figure 5). PCAs contributed to 84.96% of the variance in plant and 89.93% of the variance in soil biplots (Figure 5). In both biplots, PCA showed that wastewater application exhibited a highly negative correlation with different parameters of growth; while, wastewater application was highly positively correlated with ROS, CMP, and Cr and Pb.

In the biplot of a plant, proteins showed a highly positive relationship with CAT R (root) and APX R but a highly negative relationship with H_2_O_2_ R and MDA R. Similarly, total Cr and Pb have a highly negative relationship with protein, CAT, and APX in stem and leaf; while, total Cr and Pb positively correlated with H_2_O_2_, MDA, and CMP in root, stem, and leaf of *Solanum melongena* L. plant. The PCA results investigated that CAT, APX, proteins, and total phenolics of root, stem, and leaf are highly positively correlated with growth parameters; while, total Cr and Pb, H_2_O_2_, MDA, and CMP are highly negatively correlated with growth parameters of *Solanum melongena* L. plant. Biplots showed the maximum positive effect of ZnBc and CP-Silica gel in association with bacterial sp. (B1, B2, and B3) in the growth and development of *Solanum melongena* L. plant. However, results are well pronounced with ZnBc + B3 specifically in biochemical parameters of all parts of the plant by lowering the rates of total Cr and Pb, H_2_O_2_, MDA, and CMP in *Solanum melongena* L. plant; while CP-Silica gel + B3 was investigated as the best treatment to improve the chlorophyll content and activities of secondary metabolites in the leaf. Wastewater application has the greatest impact on physiological and biochemical parameters of the *Solanum melongena* L. plant; hence, wastewater showed a highly positive relationship with total Cr and Pb in plant and soil biplots. The PCA results significantly investigated that the application of CP-Silica gel and ZnBc along with B3 have the greatest effect on growth parameters through regulating toxicity of total Cr and Pb stress in the *Solanum melongena* L. plant.

## Discussion

### Influence of ZnBc, CP-silica gel, and bacterial sp. to improve Chl. content which stimulated growth of *Solanum melongena* L. plant under total Cr and Pb stress

The growth of *Solanum melongena* L. including its fresh and dry weights was significantly (*p* ≤ 0.01) affected by the wastewater application (control) (Table 3). It might be due to the reduced cell divisions as total Pb and Cr can bind the cell wall of a plant which directly retards the growth and development of the plant through minimum uptake of water and other nutrients through the xylem. So, the biomass of the plant was significantly (*p* ≤ 0.01) decreased (Muro-González et al., 2020). According to Kumar and Pathak (2018), physiological as well as structural changes in different plants are caused by heavy metals stress (As, Pb, Cu, Cr, and Hg). However, ZnBc and CP-silica gel along with B3 significantly (*p* ≤ 0.01) enhanced the FW and DW in all plant parts (Table 3). Silicon can increase the biomass and growth of plants by enhancing the mineral contents such as potassium, phosphorous, calcium, and amino acids (Khan et al., 2018). Similarly, an increase in biomass of different plants was examined by the applications of different rates of Zn-doped biochar under heavy metal-stressed conditions (Bruun et al., 2014; Abbas et al., 2017; Kanwal et al., 2018). As, bacterial isolates played a vital role in the growth and development of the *Solanum melongena* L. plant (Figure 5); similarly, *Bacillus subtilis* also has been reported as a plant growth promoter bacteria which significantly (*p* ≤ 0.01) reduced (>80%) Pb^2+^ from mung beans under Pb^2+^-spiked soil (Arif et al., 2019). However, the bacterial (*Pseudomonas pseudoalcaligenes* and *P. putida*) association with silicon was investigated as efficient treatment to promote growth and photosynthetic pigments in coriander under salinity stress (Al-Garni et al., 2019). Similarly, a study by Naveed et al. (2020) examined that application of biochar and gravel sand along with *Enterobacter sp*. significantly (*p* ≤ 0.01) reduced the uptake of Cd in a pea plant by increasing the pea height (47%) and its biomass (57%).

Similarly, chlorophyll contents (a and b) were significantly (*p* ≤ 0.01) decreased with wastewater application in control when compared to all other treatments; however, CP-silica gel in combination with B3 significantly (*p* ≤ 0.01) increased the chlorophyll contents (Table 3). A study reported that under heavy metals stress, enzymes in the Calvin cycle and electron transport chain altered their movement causing the destruction of thylakoid membranes, chloroplast, and stomata cells that further led to a reduced content of chlorophyll in plants (Morales et al., 2018). A similar trend was examined in our experiment (Table 3). Interestingly, ZnBc showed the best performance to regulate total Cr and Pb stress (due to its acidic nature and high binding capacities) when combined with B3 which doubled the effectiveness of amendments rather than control (Table 3). Minerals doped biochar (Mg, Zn, and Fe) were reported to enhance the chlorophyll contents in different plants such as wheat (Akhtar et al., 2015b), soya bean (Lyu et al., 2016), maize (Akhtar et al., 2015c), and potato (Akhtar et al., 2015a) by normalizing and triggering the functions of photo system II (PSII), ETC (electron transport chain), and recovery of chloroplasts (Younis et al., 2015). A similar study revealed that the application of *Bacillus sp*. and hardwood biomass alone and in association significantly (*p* ≤ 0.01) increased the photosynthetic pigments in *Arabidopsis thaliana* under As and Pb contaminated soil (Simiele et al., 2021). Likewise, a drastic decline in the chlorophyll contents was reported in coriander under Pb stress but *B. ceruse* strain 264ZG5 (KF831395) (S6) + silicon significantly (*p* ≤ 0.01) increased the content of chlorophyll a and b (Fatemi et al., 2020).

### Influence of ZnBc, CP-silica gel, and bacterial sp. to combat heavy metal stress in *Solanum melongena* L. plant under total Cr and Pb stress

The total chromium and Pb stress was significantly (*p* ≤ 0.01) regulated in soil and its minimum uptake through root and shoot of *Solanum melongena* L. plant (Figure 2). The application of ZnBc, CP-silica gel, and bacterial amendments efficiently hindered the toxicity level of Cr^6+^ and Pb^2+^ (Zhang et al., 2013; Wang et al., 2014) by decreasing their concentrations in soil and plant (Figure 2). It was also examined that the concentration of total Pb was <4 mg kg^−1^ in applied wastewater but the roots, stems, and leaves of *Solanum melongena* L. plant showed higher concentrations (Figure 2), which revealed that seedlings of *Solanum melongena* L. plant accumulated total Pb prior to pot experiment. Interestingly, total Pb was efficiently regulated and prevented further translocation from root to aerial parts by the application of CP-silica gel and ZnBc (Table 5).

**Table 5:** Bioconcentration (BCF) and translocation (TF) factors of total Cr and Pb in *Solanum melongena* L. and percent change (%) of total Cr and Pb in soil which were calculated from the mean values of total Cr and Pb analyzed from soil, root, stem, and leaf under different treatments.

**Treatm-ents**	**BCF in Root (mg/kg)**		**BCF in Stem (mg/kg)**		**BCF in Leaf (mg/kg)**		**TF (mg/kg)**	**TF (mg/kg)**	**Change of total Cr in soil** **(%)**	**Change of total Pb in soil** **(%)**
	**Cr**	**Pb**	**Cr**	**Pb**	**Cr**	**Pb**	**Cr**	**Pb**		
T1	0.18	7.78	0.15	6.33	0.13	5.34	0.88	0.81	0	0
T2	0.12	2.41	0.07	1.86	0.03	1.66	0.54	0.77	−60.9	−46.8
T3	0.11	3.57	0.06	2.92	0.03	1.99	0.55	0.81	−17.3	−52.4
T4	0.11	3.93	0.06	2.89	0.05	2.82	0.57	0.73	31.8	55.8
T5	0.10	5.15	0.07	2.91	0.04	3.58	0.76	0.56	−42.7	−5.10
T6	0.09	3.18	0.08	2.63	0.03	1.91	0.88	0.82	−29.8	−15.8
T7	0.10	1.96	0.06	0.72	0.02	0.91	0.62	0.37	70.3	12.0
T8	0.07	1.59	0.05	1.17	0.02	0.63	0.74	0.73	−13.3	−11.0
T9	0.06	0.85	0.04	0.50	0.02	0.15	0.65	0.58	−26.3	−37.4
T10	0.08	2.58	0.04	1.54	0.02	0.92	0.54	0.59	47.1	21.3
T11	0.07	1.25	0.03	0.27	0.01	0.17	0.53	0.22	−40.2	−51.6
T12	0.04	0.67	0.03	0.08	0.01	0.05	0.65	0.11	−31.4	−25.3

Similarly, reduced concentrations of heavy metals in plants were reported due to their immobilization by binding at large surface areas of chitosan and rice husk biochar, which efficiently lower the uptake of heavy metals from the root and its translocation toward the shoot (Ramzani et al., 2017; Tripathi et al., 2017; Turan et al., 2018a; Hussain et al., 2019). Metallic ions are chelated and immobilized by intra-molecular bindings due to the presence of hydroxyl and amino groups in CP-Silica gel (Tripathi et al., 2017). A similar study revealed that chitosan and rice husk biochar significantly (*p* ≤ 0.01) reduced the concentrations of heavy metals such as Cr, Pb, Co, Cu, and Cd from the *Solanum melongena* L. plant by analyzing its root, shoot, and fruit (Turan et al., 2018a). Another study revealed that the application of pine-wood biochar and *Bacillus* sp. efficiently decreased the concentrations of Cr, Cu, Pb, and Co by 3.11- to 17-folds in the *Hordeum vulgare* L. plant (Andrey et al., 2019). Likewise, the application of silicon along with *Enterobacter cloacae* and *Bacillus drentensis* significantly (*p* ≤ 0.01) combats abiotic stresses (such as heavy metals and drought stress) in mung bean plants (Etesami, 2018).

Bioconcentration (BCF) and translocation factors (TF) were investigated in the proposed study (Table 5). As, BCF >1 can be found for total Pb in roots, stems, and leaves (Table 5). More than 50% accumulation was examined for total Cr in the root (0.12 mg kg^−1^) and stem (0.08 mg kg^−1^), and the remaining concentrations were <50%. Translocation factors (TF) of total Cr and Pb can also be seen in Table 5. Based on this table, TF from root to shoot was efficiently inhibited as the concentrations of total Cr and Pb (TF) were <1. These findings indicated that more total Cr and Pb ions accumulate in roots rather than shoots of the *Solanum melongena* L. plant. TF factor is also based on the influence of capillary action of the *Solanum melongena* L. plant for the translocation of total Cr and Pb (Takarina and Pin, 2017).

A similar study examined decreased BCF and TF in the stem and leaf of the plant rather than root; because more toxins accumulate in roots which are inhibited from further translocating into aerial parts of the plant in the presence of adsorbing agents (Takarina and Pin, 2017). It was examined that the adsorbing and chelating properties of CP-silica gel and ZnBc when combined with B3 effectively controlled the accumulation and translocation of total Cr and Pb ions in the *Solanum melongena* L. plant as compared with control (Table 5). The scenario of percent change (%) variance showed that concentrations of total Cr and Pb significantly reduced in soil under all treatments except control (Table 5). Negative signs indicated minimum concentrations of total Cr and Pb present in the soil as compared with other treatments. Results were more pronounced by comparing the scenario of BCF and TF with a similar study conducted by Takarina and Pin (2017).

### Influence of ZnBc, CP-silica gel, and bacterial species on reactive oxygen compounds and antioxidant defense production in *Solanum melongena* L. plant under total Cr and Pb stress

The high concentration of H_2_O_2_, MDA, and CMP was examined in control as compared with all other treatments (Table 4). It was examined that the addition of Zn-doped biochar and CP-Silica gel with bacterial species played a vital role in scavenging ROS by triggering the activities of antioxidant enzymes (CAT and APX) by increasing the number of hydroxyl groups (Figure 3). Under heavy metals stress conditions, burst production of ROS disturbs biochemical mechanisms in plants which leads to lipid peroxidation and CMP that further cause damage to the plasma membrane (Dad et al., 2021). However, soil Cd stress was better controlled with Fe-doped biochar application and an increase in the growth and physiology of radish reported by Dad et al. (2021). Naeem et al. (2022) also reported a reduction in Pb^2+^ and Cr^6+^ concentration with Fe-doped biochar application which leads to reduced MDA production in the tomato plant. Similarly, applications of silicon significantly reduced MDA production in soybean plant under salt-stressed conditions (Osman et al., 2021). A similar study by Shah et al. (2021) investigated the decreased level of ROS (MDA and H_2_O_2_) production by activating antioxidant enzymes in the *Solanum melongena* L. plant through the application of *Bacillus subtilis* and silicon. Likewise, toxic effects of heavy metals (Cr, Pb, Cu) were significantly (*p* ≤ 0.01) reduced by the application of *Alcaligenes faecalis* (MG257493.1), *Bacillus cereus* (MG257494.1), and *Alcaligenes faecalis* (MG966440.1) alone and in association with ZnO nanoparticles through triggering the activities of antioxidant enzymes in sorghum (El-Meihy et al., 2019; Siddiqui et al., 2019).

Catalase (CAT) activities were enhanced in mung beans with silicon applications by controlling lipid peroxidation (Ahmed et al., 2019). CP-Silica gel has been reported to enhance essential nutrients in plants by adjusting the osmotic stress of the cell and limiting free radicle accumulation (Wang et al., 2016). Similarly, APX activities were also significantly (*p* ≤ 0.01) triggered with applications of ZnBc, CP-Silica gel, and bacterial (B1, B2, and B3) amendments under total Cr and Pb stress (Figure 2). The findings of this study are also supported by Turan et al. (2018b), which demonstrated that APX activities were highly triggered in sunflower leaves by biochar and silicon applications. The toxic effects of H_2_O_2_ can be offset by the efficient activities of APX in different organelles (such as chloroplast, cytosol, peroxisome, and mitochondria) of plants under organic and inorganic amendments (Aziz et al., 2016). Thus, it can be suggested that ZnBc and CP-Silica gel can be used to balance redox homeostasis (Aziz et al., 2016) in plants. The literature revealed that studies on chitosan and rice husk biochar also found the same results of an antioxidant defense mechanism by scavenging ROS in sunflower (Turan et al., 2018a), and mung bean (Ramzani et al., 2017) under Cu, Cr, and Pb stress.

### Influence of ZnBc, CP-silica gel, and bacterial sp. on secondary metabolites in *Solanum melongena* L. plant under total Cr and Pb stress

The concentrations of protein and total phenolics were significantly (*p* ≤ 0.01) improved by the applications of ZnBc, CP-Silica gel, and bacterial sp. under wastewater application (Figure 4). Results were well pronounced with ZnBc and B3 to combat total Cr and Pb stress and stimulate the activities of secondary metabolites (Figure 4). Biochemical compounds (carbohydrates, lipids, proteins, nucleic acids, and phenolics) also have been reduced in previous studies, for example, in the *Solanum melongena L*. plant (Kumar, 2015; Kumar et al., 2016), maize (Abou-Hassan et al., 2018) and spinach (Saini et al., 2015) under heavy metals stress due to the enhanced rate of protein denaturation. A similar study investigated that the applications of Zn-doped biochar and nano-particles efficiently improved the rate of protein synthesis under heavy metals stress with the stimulation of the plant's immune system (Farooq et al., 2020; Shahhoseini et al., 2020). Zinc efficiently stimulates the synthesis of secondary metabolites by increasing the number of secret trichomes which activate the protein synthetases in the pathway of chlorophyll biosynthesis. Thus, the pathway of chlorophyll synthetases is efficiently protected from the damaging effects of free radicles (Shahhoseini et al., 2020). Likewise, plant proteins can also be enhanced by applying silicon in Pb-stressed soil (Kiran and Prasad, 2019). Another study suggested that the application of biochar and gravel sand along with *Enterobacter* sp. significantly increased (41%) the protein content in pea plant under Cd-stressed soil (Naveed et al., 2020). Similarly, protein content was efficiently increased in the *Solanum melongena* L. plant through the application of *Bacillus subtilis* associated with silicon under Pb contaminated soil (Shah et al., 2021).

It was also examined that the concentration of total phenolics was increased than that of proteins which indicated more regulation of total Cr and Pb stress because phenolics are involved in ROS scavenging (Figure 4). The improvement in biochemical compounds of plants might be due to the increase in the water-holding capacity of soil with the applications of chitosan and rice husk biochar (Pandey and De, 2017; Pituya et al., 2017; Suliman et al., 2017). Therefore, the production of starch, amino acids, proteins, phenolics, carotenoids, and chlorophyll content is enhanced by the improvement of the plant's metabolic activities due to the improved flow of water through the xylem toward the leaves (Younis et al., 2015; Hafeez et al., 2017). Another study suggested that the application of silicon along with *Pseudomonas pseudoalcaligenes* and *P. putida* significantly (*p* ≤ 0.01) increased the concentration of total phenolics in coriander under salinity-stressed conditions (Kanwal et al., 2018). Similarly, Andrey et al. (2019) examined the significant (*p* ≤ 0.01) effect of pine-wood biochar and *Bacillus* sp. association on the growth and development (activities of primary and secondary metabolites) of *Hordeum vulgare* L. plant under heavy metals (Cr, Cu, Pb, Co, Ni, Mn, and Zn) stressed soil. In developing countries like Pakistan, Zn remained the most deficient micronutrient in soil; therefore, the application of ZnBc with suitable microbial sp. such as *Bacillus subtilis* in contaminated areas would not only enhance the fertility of the soil but also reduce the toxic impacts of heavy metals on crops grown in such soils.

## Conclusion

CP-Silica gel with B3 showed efficient performance to combat total Cr and Pb stress through scavenging burst production of ROS and stimulating the enzymatic and non-enzymatic defense mechanisms of the *Solanum melongena* L. plant which ultimately led to enhanced growth and development of this plant. Similarly, zinc-enriched biochar in combination with B3 also proved to be the best treatment in regulating *Solanum melongena* L. growth and defense mechanism. It not only reduced the Cr and Pb toxicity in soil but also reduced their translocation to different plant parts. Hence, applications of ZnBc with the interaction of bacterial isolates proved to be an eco-friendly soil conditioner that can be used in developing countries to limit the deleterious effects of total Cr and Pb pollution.

## Data Availability Statement

The original contributions presented in the study are included in the article/Supplementary material, further inquiries can be directed to the corresponding author/s.

## Author contributions

W-u-DK conceived the idea and designed the research. UR, W-u-DK, and MA conducted the experiment and analyzed the data. UR, W-u-DK, MS, and MF developed the full draft and analyzed the data. W-u-DK, MF, and MS revised and critically reviewed the manuscript. All authors contributed to the subsequent development, approved the final manuscript, and reviewed the manuscript carefully.

## Funding

The authors are highly thankful to the Punjab Agricultural Research Board (PARB) of Pakistan for financing this research under the Competitive Grant Program, Project# 894. Also, this study was supported by the Researchers Supporting Project Number (RSP-2021/194), King Saud University, Riyadh, Saudi Arabia.

## Conflict of interest

The authors declare that the research was conducted in the absence of any commercial or financial relationships that could be construed as a potential conflict of interest.

## Publisher's note

All claims expressed in this article are solely those of the authors and do not necessarily represent those of their affiliated organizations, or those of the publisher, the editors and the reviewers. Any product that may be evaluated in this article, or claim that may be made by its manufacturer, is not guaranteed or endorsed by the publisher.
