# Supplementary figures and images for: Comparative efficiency of silica gel, biochar, and plant growth promoting bacteria on Cr and Pb availability to Solanum melongena L. in contaminated soil irrigated with wastewater
Source: Front Plant Sci. 2022 Aug 4;13:950362. doi: 10.3389/fpls.2022.950362 (PMC9386531; doi:10.3389/fpls.2022.950362)

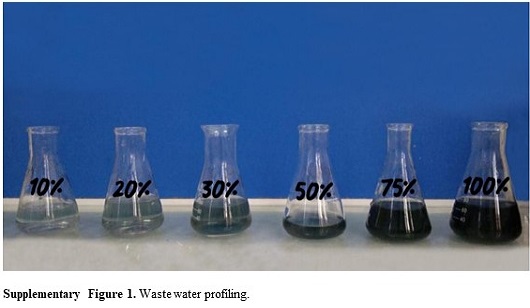

Supplement: Supplementary file 1 [file Image_1.jpeg]
